# Cepstral Peak Prominence Smoothed - CPPS and Acoustic Voice Quality Index - AVQI in healthy and altered children's voices: comparation, relationship with auditory-perceptual judgment and cut-off points

**DOI:** 10.1590/2317-1782/20242023047en

**Published:** 2024-05-27

**Authors:** Evelyn Carla dos Santos Rabelo, Ana Paula Dassie-Leite, Vanessa Veis Ribeiro, Glaucya Madazio, Mara Suzana Behlau

**Affiliations:** 1 Centro de Estudos da Voz – CEV - São Paulo (SP), Brasil.

**Keywords:** Voice, Children, Acoustics, Cepstral Peak, Vocal Quality

## Abstract

**Purpose:**

To compare the acoustic measurements of Cepstral Peak Prominence Smoothed (CPPS) and Acoustic Voice Quality Index (AVQI) of children with normal and altered voices, to relationship with auditory-perceptual judgment (APJ) and to establish cut-off points.

**Methods:**

Vocal recordings of the sustained vowel and number counting tasks of 185 children were selected from a database and submitted to acoustic analysis with extraction of CPPS and AVQI measurements, and to APJ. The APJ was performed individually for each task, classified as normal or altered, and for the tasks together defining whether the child would pass or fail in a situation of vocal screening.

**Results:**

Children with altered APJ and who failed the screening had lower CPPS values and higher AVQI values, than those with normal APJ and who passed the screening. The APJ of the sustained vowel task was related to CPPS and AVQI, and APJ of the number counting task was related only to AVQI and CPPS numbers. The cut-off points that differentiate children with and without vocal deviation are 14.07 for the vowel CPPS, 7.62 for the CPPS numbers and 2.01 for the AVQI.

**Conclusion:**

Children with altered voices, have higher AVQI values and lower CPPS values, when detected in children with voices within the normal range. The acoustic measurements were related to the auditory perceptual judgment of vocal quality in the sustained vowel task, however, the number counting task was related only to the AVQI and CPPS. The cut-off points that differentiate children with and without vocal deviation are 14.07 for the CPPS vowel, 7.62 for the CPPS numbers and 2.01 for the AVQI. The three measures were similar in identifying voices without deviation and dysphonic voices.

## INTRODUCTION

Change in vocal quality is considered a common symptom in childhood; the occurrence of vocal problems varies between 6 and 37% in the pediatric population^(1,2)^. It can negatively impact the child's life, in their overall health, socio-educational development, communicative efficiency, and participation in school activities^(1,3)^. Its etiology is multifactorial, as it can be organic, behavioral, or related to emotional factors^(1,4)^.

One challenge in pediatric vocal clinics is the anatomophysiological differences between children and adults. The pediatric larynx exhibits neuromuscular immaturity, with a glottic proportion of 1.0; the layers of the vocal fold lamina propria are undifferentiated, and the vocal ligament is immature. Tissues are vascularized, with a tendency for edema, and the vocal tract is shortened^(5-8)^. Additionally, due to developmental factors in children, the presence of instability, breathiness, and roughness are expected without indicating vocal disorder^(5-8)^.

Vocal assessment in the speech-language pathology clinic is multidimensional^(9)^. The recommended procedures include self-assessment and parent evaluation, perceptual-auditory judgment (PAJ), aerodynamic evaluation, visual and structural analysis of the larynx, and acoustic analysis^(9)^.

Acoustic analysis has been evolving, with a noticeable shift from traditional measures such as frequency perturbation indices (jitter and shimmer) towards more robust measures, like the Acoustic Voice Quality Index (AVQI). The AVQI is a multiparametric measure that evaluates various acoustic and cepstral measures that do not necessarily depend on fundamental frequency extraction, such as the Central Peak Prominence Smoothed (CPPS)^(5,10,11)^. Considering that voice should be assessed in a multidimensional manner, extracting isolated parameters may be insufficient for its characterization^(12)^. Currently, CPPS stands as the primary acoustic measure endorsed for vocal clinical evaluation by the American Speech-Language-Hearing Association (ASHA)^(9)^.

The CPPS evaluates the periodicity of vocal emissions and represents a variation of the Cepstral Peak Prominence (CPP). This measure has high accuracy and stable extraction, allowing the analysis of sustained vowel emission and the continuous speech signals. CPPS smooths short-term fluctuations in the signal and aims to identify the presence of the cepstral peak, which is a crucial marker of emission periodicity. CPPS is expressed in decibels (dB) and is utilized in voice and speech research, along with clinical settings for assessing vocal disorders, thereby enhancing diagnostic precision and efficiency^(13-16)^.

On the other hand, AVQI is a multiparametric index; hence, it utilizes various parameters to provide a single score for the vocal quality. It considers sustained vowel and continuous speech, counting numbers in Brazilian Portuguese, to offer higher ecological validity in the vocal assessment^(12)^. Moreover, the AVQI has demonstrated good diagnostic accuracy^(15)^, making it a suitable tool for determining the presence of vocal disorders.

The speech tasks employed for PAJ in vocal clinics include sustained emission, continuous speech, and spontaneous emissions^(17)^. In the adult population, sustained emission may be perceived as more deviated than speech tasks^(18)^. Therefore, the voice of an individual may vary depending on the type of vocal task requested^(19)^.

These measures hold significant clinical relevance, being considered an important component of vocal clinical assessment with children. Currently, there is a lack of Brazilian Portuguese studies determining whether, for pediatric voices, considering their full complexity, these metrics can effectively differentiate between children with normal and deviated vocal quality, and whether any particular vocal task has greater relevance for this discrimination. Additionally, understanding the correlation with PAJ and establishing cutoff points to facilitate the clinical interpretation of CPPS and AVQI values within the pediatric voices is imperative.

Therefore, the research aims to compare the acoustic metrics of CPPS and AVQI in children with normal and deviated vocal quality, correlate them with PAJ, and establish cutoff points distinguishing these two categories.

## METHODS

This is an observational, cross-sectional, and analytical study. The study was approved by the Ethics Committee of the institution under the number 2179.073/2010-03. Parents or legal guardians of the children signed the Informed Consent Form. Children 12 years old also signed the Informed Assent Form.

The sample included data and vocal recordings of participants extracted from a pre-existing database obtained in a previous study. These participants were children recruited from a university-affiliated hospital within a federal university, while they awaited elective medical appointments in various pediatrics specialties (such as general, dermatology, and endocrinology, among others) through the Brazilian Unified Health System (*Sistema Único de Saúde*, known as SUS). The sample was collected conveniently to explore the voices of children in the general population.

The inclusion criteria for this study were: prepubertal children, confirmed through medical evaluation regarding pubertal staging; both sexes; minimum age of three years. Exclusion criteria were: colds or acute airway obstructions for any reason on the day of data collection; auditory complaints; history of other health problems impacting voice. A total of 185 children were eligible for the study, 93 boys and 92 girls, aged between 3 and 12 years (mean 6.86 ± 2.2).

The vocal material used from the voice database comprised the sustained vowel “é” and counting numbers 1 to 10, at habitual pitch and loudness. The samples were recorded using the VOXMETRIA® program (CTS Informática, version 2.5) in wave sound file format, on a Dell® laptop, with a head-mounted unidirectional microphone, Karsect model HT-9, connected to the Andrea Pure Audio sound interface. The microphone was positioned approximately one centimeter from the corner of the participant's mouth (diagonal position). If there were difficulties in calibration regarding the loudness presented by the child (too strong or too weak), the microphone was repositioned where the gain was appropriate, comprising a sufficient signal level, around two-thirds of the audio window, as indicated in the VOXMETRIA® program (CTS Informática, version 2.5).

For this research, the audio files extracted from the database were edited by removing the initial and final segments of the vowel sample recordings, which typically correspond to a period of natural voice instability, while retaining the middle segment, which lasted between three and four seconds. The samples underwent editing to eliminate the silent segments between emissions in the counting numbers samples.

The audio files were recorded using the Audacity program, and due to slight differences in signal intensity during the recording of both vowels and numbers, the samples were standardized through manual calibration in the Audacity® program (version 2.0.3). To calibrate the voice signals, X was considered as the absolute maximum value of each signal (or the infinity norm of the vector containing the signal samples). Next, each signal sample was multiplied by the reciprocal of X (or 1/X). Since each sample can take values between -1 and +1, where 1 is associated with the maximum volume level, this procedure linearly amplifies the signal to only increase its volume without altering its spectral and temporal properties.

Subsequently, the vocal samples underwent acoustic analysis for the CPPS and AVQI extraction using the PRAAT software (version 6.06). For the CPPS extraction, specific commands for obtaining measurements in Brazilian Portuguese (BP) speakers were utilized, conducting one extraction for the vowel task and another for the counting numbers task. The AVQI 03.01 Praat script was used to obtain the AVQI value, concurrently processing samples from both the vowel and counting numbers tasks^(13)^.

The PAJ was conducted by three speech-language pathologists and voice specialists, each with over ten years of clinical and scientific experience in the voice field, and with expertise in pediatric dysphonia. They were not previously familiar with the research objectives. The judges collectively analyzed the samples, ensuring consensus by convening in the same environment under identical conditions: a quiet setting with playback on a professional-quality speaker. Samples from both vowels and counting numbers were analyzed separately. Judges listened to each sample repeatedly as needed, assigning an overall degree of vocal deviation (G) using a four-point numerical scale: zero for no deviation, one for mild deviation, two for moderate deviation, and three for severe deviation. The PAJ session lasted approximately two hours and was conducted on a single day. The internal agreement of judges' consensus-based analysis was assessed using the Kappa test, with approximately 10% of the samples repeated, yielding a Kappa value of 0.75.

For the data analysis in this study, the vocal samples were classified as follows: samples rated by the judges as having no deviations (zero) or mild deviations (one) were considered normal; samples rated by the judges as having moderate (two) or severe (three) deviations were considered altered. Subsequently, to establish a single outcome for each child, reflecting a potential vocal screening scenario, the following procedure was employed: children with normal voices in both tasks (vowel and counting number), or with only one task classified as altered, were categorized as passing in a vocal screening scenario; children with altered voices in both tasks (vowel and counting number), were categorized as failing in a vocal screening scenario. It is noteworthy that all children in the sample exhibited a maximum difference of one grade between both speech tasks.

The data were analyzed descriptively and inferentially. The SPSS 25.0 software was used. In the descriptive analysis of quantitative variables, measures of central tendency (mean and median), variability (standard deviation), and position (minimum, maximum, first and third quartiles) were calculated. For descriptive analysis of nominal qualitative variables, absolute frequency and relative frequency percentages were computed.

For the present study, the acoustic measures served as dependent variables, while PAJ and vocal screening were the independent variables. A comparison of acoustic measures based on PAJ and vocal screening was performed using the Mann-Whitney test. Predictive analysis of acoustic measures was conducted through multiple linear regression analysis, using the backward method. Additionally, ROC curve analyses were conducted for the acoustic measures, using the vocal screening result as the gold standard. The significance level was set at 5%.

## RESULTS

The vowel task presented a higher occurrence of altered voices (n=105; 56.76%) in comparison to normal voices (n=80; 43.24%). The counting numbers presented 72 altered voices (38.92%) and 113 normal voices (61.08%). The screening outcome showed a higher frequency of normal voices (normal n=128, 69.19%; altered n=57, 30.81%).

Children identified with vocal deviations based on the PAJ assessments for vowel and number tasks exhibited lower CPPS values (vowel - p<0.001; p=0.044; numbers p=0.001; p<0.001) and higher AVQI values (p<0.001; p<0.001), compared to children without vocal deviations according to the PAJ (Tables 1 and 2).

**Table 1:** Comparison of dependent variables based on the independent variable PAJ classification for vowel task

Variable	PAJ Vowel	Mean	SD	Minimum	Maximum	1Q	Median	3Q	p-value
CPPS vowel	Normal	14.71	1.70	10.50	19.01	13.36	14.61	15.75	0.000
	Altered	12.95	1.61	9.03	17.76	11.87	12.66	14.22	
CPPS number	Normal	8.34	1.42	5.78	12.45	7.28	8.28	9.19	0.001
	Altered	7.63	1.26	4.95	10.68	6.96	7.53	8.36	
AVQI	Normal	1.24	0.98	-1.70	5.04	0.69	1.23	1.78	0.000
	Altered	2.06	0.89	-0.11	4.02	1.58	2.15	2.65	

Mann-Whitney Test

Caption: PAJ = perceptual-auditory judgment; SD = standard deviation; 1Q = first quartile; 3Q = third quartile

**Table 2:** Comparison of dependent variables based on the independent variable PAJ classification for the number task

Variable	PAJ Numbers	Mean	SD	Minimum	Maximum	1Q	Median	3Q	p-value
CPPS vowel	Normal	13.93	1.93	9.36	19.01	12.48	14.11	15.00	0.044
	Altered	13.37	1.71	9.03	17.76	12.24	13.13	14.47	
CPPS numbers	Normal	8.29	1.31	5.78	12.45	7.28	8.17	9.22	0.000
	Altered	7.38	1.29	4.95	11.65	6.62	7.30	8.12	
AVQI	Normal	1.46	0.99	-1.70	5.04	0.77	1.46	2.14	0.000
	Altered	2.08	0.93	-0.63	4.02	1.55	2.21	2.69	

Mann-Whitney Test

Caption: PAJ = perceptual-auditory judgment; SD = standard deviation; 1Q = first quartile; 3Q = third quartile

Children who did not pass the vocal screening exhibited lower CPPS values (vowel and numbers = p<0.001) and higher AVQI values (p<0.001) compared to children who passed the screening (Table 3).

**Table 3:** Comparison of dependent variables based on the independent variable vocal screening

Variable	Screening	Mean	SD	Minimum	Maximum	1Q	Median	3Q	p-value
CPPS vowel	Pass	14.03	1.91	9.36	19.01	12.80	14.12	15.15	<0.001
	Fail	12.99	1.54	9.03	17.76	12.02	12.66	14.31	
CPPS number	Pass	8.24	1.35	5.78	12.45	7.25	8.15	9.18	<0.001
	Fail	7.25	1.17	4.95	10.06	6.57	7.24	8.08	
AVQI	Pass	1.44	0.99	-1.70	5.04	0.72	1.45	2.14	<0.001
	Fail	2.31	0.78	0.10	4.02	2.05	2.34	2.74	

Mann-Whitney Test

Caption: SD = standard deviation; 1Q = first quartile; 3Q = third quartile

Three predictive models were developed to determine if the independent variables PAJ for vowel and number were predictive of the dependent variables AVQI, CPPS vowel, and CPPS number (Table 4). The independent variables PAJ for vowel (β = 0.338; t = 4.724; p = 0.000 ; β = -0.164; t = -2.208; p = 0.029) and PAJ for number (β = 0.177; t = 2.474; p = 0.014 ; β = -0.262; t = -3.524; p = 0.001; respectively) were predictive of the dependent variables AVQI [F(2, 182) = 21,200; p <0.001; R2 = 0.189] and CPPS [F(2, 182) = 13,174; p <0.001; R2 = 0.117]. The independent variable PAJ for vowel (β = -0.471; t = -7.219; p <0.001) was predictive of the dependent variable CPPS vowel [F(1, 183) = 52.119; p<0.001; R2 = 0.222].

**Table 4:** Predictive model of the dependent variables AVQI, vowel CPPS, and number CPPS

Model		Unstandardized coefficients		Standardized coefficients	t	p-value
		B	Standard Error	Beta		
AVQI	(Constant)	1.172	0.106		11.031	0.000
	Vowel	0.689	0.146	0.338	4.724	0.000
	Number	0.367	0.148	0.177	2.474	0.014
CPPS vowel	(Constant)	14.715	0.185		79.742	0.000
	Vowel	-1.768	0.245	-0.471	-7.219	0.000
CPPS number	(Constant)	8.481	0.150		56.621	0.000
	Number	-0.737	0.209	-0.262	-3.524	0.001
	Vowel	-0.454	0.206	-0.164	-2.208	0.029

Multiple linear regression, stepwise method

Independent variables: vowel, numbers

Caption: t = T statistic

**Figure 1:**
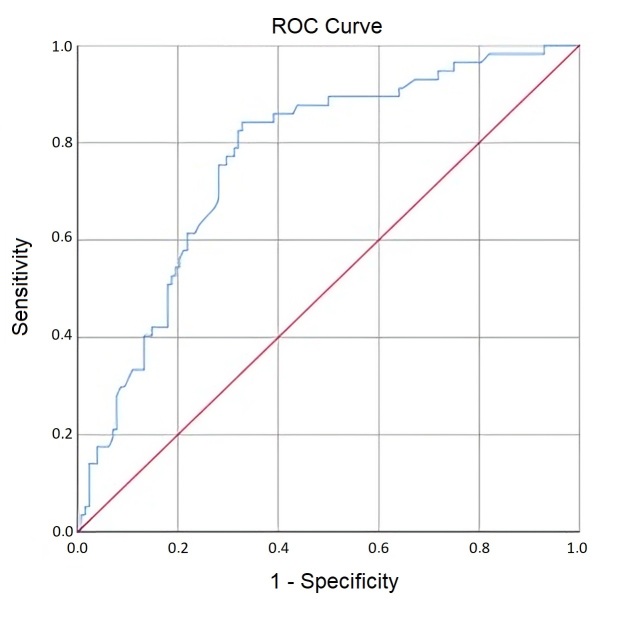
AVQI ROC Curve

The results present a statistically significant curve (Figure 1) for AVQI (AUC = 0.76; SE = 0.04; p < 0.001; 95% CI = 0.69 – 0.84), indicating that 76% of cases failing the vocal screening have higher AVQI values compared to those passing the screening, when chosen randomly. The cutoff point maximizing sensitivity and specificity was 2.05 (i.e., below 2.0 and above 2.1), with sensitivity of 0.75 and specificity of 0.72. AVQI exhibits a similar capacity in identifying both deviated and non-deviated voice qualities.

**Figure 2:**
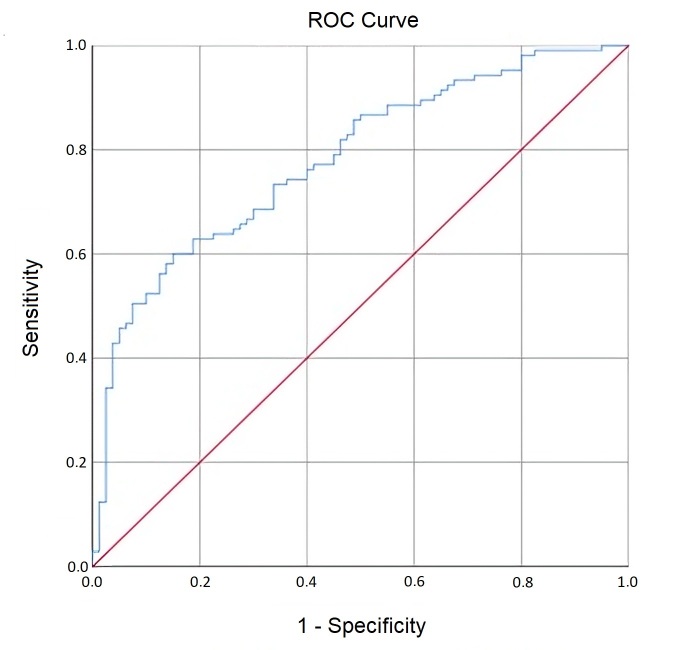
CPPs ROC Curve for Vowel

A statistically significant curve was also identified for CPPS vowel (Figure 2) (AUC = 0.78; SE = 0.03; p < 0.001; 95% CI = 0.715 - 0.846), revealing that when selected randomly, 78% of cases with vocal deviation in the PAJ of the vowel sample displayed lower CPPS vowel values compared to cases without vocal deviation. The cutoff point that maximized sensitivity and specificity was 14.07 (i.e., below 14.0 and above 14.1), with a sensitivity of 0.73 and specificity of 0.66. CPPS vowel demonstrates a similar capacity in identifying deviated and non-deviated voice qualities.

**Figure 3:**
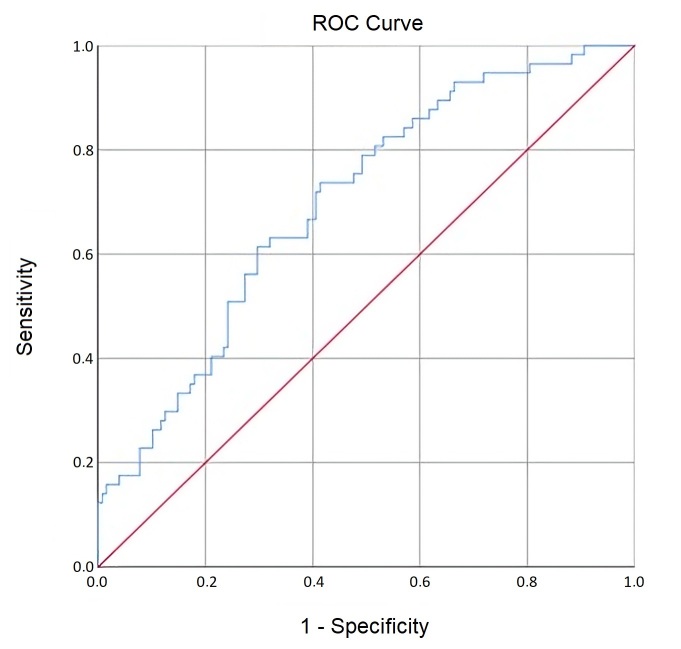
CPPs ROC Curve for Numbers

A statistically significant curve was also obtained for CPPS numbers (Figure 3) (AUC = 0.70; SE = 0.04; p < 0.001; 95% CI = 0.62 - 0.78), indicating that when chosen randomly, 70% of cases with vocal deviation in the PAJ of the counting numbers displayed lower CPPS values compared to cases without vocal deviation. The cutoff point that maximized sensitivity and specificity was 7.62 (i.e., below 7.6 and above 7.7), with a sensitivity of 0.63 and specificity of 0.68. CPPS numbers have a similar ability to identify voices with and without deviation.

## DISCUSSION

The relative frequencies of deviated voices in the PAJ were high, considering a vocal screening scenario (30.81%), which involved the analysis of both sustained and continuous speech emissions. Nevertheless, these values are consistent with the literature regarding the occurrence of vocal problems in the pediatric population^(1,2).^

There was a higher occurrence of deviation in the vowel task (n=105; 56.76%) in comparison to normal voices (n=80; 43.24%). For the counting task, there were 72 altered voices (38.92%) and 113 normal voices (61.08%). The screening task showed a higher frequency of normal voices (normal n=128, 69.19%; altered n=57, 30.81%).

The AVQI and CPPS measures were observed to distinguish between children with normal and altered voices as assessed by PAJ, irrespective of whether the sample included sustained speech, continuous speech, or both. These findings align with existing literature^(9,14,15)^, suggesting that healthy children may exhibit higher CPPS values and lower AVQI values compared to those with altered voice quality.

Through the analysis of regression models used in this study, it was possible to conclude that PAJ for sustained vowels is related to all three measures, while PAJ for counting numbers is related only to AVQI and CPPS numbers. Vocal tasks are recognized to introduce variability into PAJ, with counting numbers revealing temporal and spectral fluctuations attributed to factors such as word onset and termination, pauses, voiceless phonemes, phonetic context, prosody, pitch and loudness variations, speech rate, among others. On the other hand, sustained vowels typically exhibit relatively stable subglottic and supraglottic conditions^(19)^.

With children undergoing screening, those with laryngeal alterations tend to fail in both sustained and continuous speech emissions, unlike children with normal larynges, who may only fail in one of the tasks occasionally^(7)^. In adolescent voices, sustained vowels are capable of identifying the vocal instabilities typical of the voice change period, which are not observed in counting numbers or text reading tasks^(18)^.

It is hypothesized that children who exhibit instability, anticipated as a deviation due to neuromuscular immaturity^(20)^, and breathiness associated with a posterior glottic gap, primarily resulting from the typical anatomical configuration of the cricoid cartilage in this age group^(6)^, will show increased sensitivity to vocal deviations during the vowel task. Consequently, the vowel task may serve as a predictor for all multiparametric acoustic measures. Furthermore, the results obtained from the ROC curve analysis for the AVQI and CPPS vowel variables support findings from studies conducted with children, which demonstrate the utilization of cepstral measures as indicators of vocal deviation^(9,15)^.

The AVQI demonstrated higher sensitivity values, unlike a previous study involving pediatric voices where the index exhibited higher specificity values^(15)^. However, this prior research^(16)^ established a cutoff value of 3.46, which was higher than the value obtained in the present study (2.05), thus enabling the exclusion of a larger number of children without vocal deviation.

There is an influence of the spoken language on defining cutoff measures^(21)^, and it's noteworthy that in the adult Brazilian Portuguese-speaking population, where the cutoff value is 1.33^(12)^, these values are also lower compared to populations in other countries. This emphasizes that linguistic and cultural factors may influence these measures^(22)^.

The higher cutoff value in children compared to Brazilian Portuguese adult speakers can be attributed to the expected vocal quality deviations in pediatric individuals due to the developmental process itself^(23)^, which requires different parameters and analyses^(24)^. Furthermore, this aligns with the outcome of a recent study conducted across pediatric, adult, and elderly populations within the same country, which indicated that AVQI cutoff values distinguishing normal and deviated voices are higher in the pediatric population than those observed in the adult population^(24)^.

Regarding CPPS, unlike the present study, previous research observed higher sensitivity in CPP numbers to differentiate normal and deviated voices^(9)^. Hence, this metric is likewise impacted by the linguistic nuances of diverse languages, thereby complicating the comparison of populations across various countries.

Concerning the cutoff values obtained in the present study for the differentiation of normal and altered child voices (CPPS vowel = 14.07; CPPS numbers = 7.62), previous studies have also reported higher CPP values for vowel tasks compared to counting numbers tasks^(25,26)^. No studies that established CPPS cutoff values for children were found. However, research evaluating Brazilian Portuguese children and adolescents aged between 5 and 18 years (128 girls and 131 boys) obtained mean CPPS values similar to those of the present study^(27)^. This previous study divided children and adolescents by age and sex. The age groups ranging from 5 to 11 years old presented a mean CPPS value for vowels ranging from 13.994 to 15.203. Furthermore, a study conducted using a similar measure, the CPP, concluded that cepstral measures are useful in differentiating between healthy and dysphonic children^(9)^.

The CPPS vowel and numbers cutoff values obtained in the present study are also lower than values commonly obtained in the adult population^(28)^. As previously mentioned, this is expected, once children typically exhibit vocal deviations as part of their developmental process^(26)^.

One of the present study's limitations is regarding the material of the counting numbers task, which ranged from 1 to 10, while the AVQI-validated version of Brazilian Portuguese recommends counting numbers from 1 to 11. However, the influence of the smaller counting range is not a relevant factor^(12)^.

Another limitation is the lack of subdivision of children by age group. Although some studies suggest that there are no changes in the acoustic measures’ outcomes regarding disturbance and noise with advancing age in children^(29,30)^, recent studies on cepstral measures with children and adolescents in a broader age range have revealed some differences related to gender^(27)^ and type of task^(14)^.

In this regard, given the complexity of childhood vocal development, it is suggested that future studies consider this subdivision to elucidate the distribution of CPPS and AVQI measures. However, only children proven to be pre-pubescent participated in this study; they were evaluated by a pediatrician on the day of vocal data collection. Since this study represents the first investigation into cepstral measures and multiparametric indices in children, we chose to provide comprehensive data for pre-pubertal children without further age subdivision.

The data generated from this study may serve as a valuable purpose in characterizing and interpreting the acoustic values observed in clinical practice and research involving children. Furthermore, both AVQI and CPPS can serve as objective parameters for evaluating and monitoring therapeutic progress in pediatric populations undergoing speech-language pathology interventions.

## CONCLUSION

Regardless of the speech task, children with vocal deviation presented higher AVQI values and lower CPPS values than children without vocal deviation. PAJ for the sustained vowel task predicted all acoustic measures, while PAJ for counting numbers was predictive only of the measures extracted from AVQI and CPPS. The cutoff points distinguishing children with and without vocal deviation are 14.07 for CPPS vowel, 7.62 for CPPS number, and 2.01 for AVQI. All three measures were similar in identifying voices with and without deviation.
