# Supplementary material for: Cepstral Peak Prominence Smoothed - CPPS and Acoustic Voice Quality Index - AVQI in healthy and altered children's voices: comparation, relationship with auditory-perceptual judgment and cut-off points
Source: Codas. 2024 May 27;36(4):e20230047. doi: 10.1590/2317-1782/20242023047en (PMC11189156; doi:10.1590/2317-1782/20242023047en)
Supplement: Translation

# Translation: Cepstral Peak Prominence Smoothed - CPPS e Acoustic Voice Quality Index - AVQI em vozes infantis saudáveis e alteradas: comparação, relação com julgamento perceptivo-auditivo e pontos de corte

**DOI:** 10.1590/2317-1782/20242023047pt

**Authors:** Evelyn Carla dos Santos Rabelo, Ana Paula Dassie-Leite, Vanessa Veis Ribeiro, Glaucya Madazio, Mara Suzana Behlau

**Affiliations:** 1 Centro de Estudos da Voz – CEV - São Paulo (SP), Brasil.

**Keywords:** Voz, Crianças, Acústica, Pico Cepstral, Qualidade Vocal

## Abstract

**Objetivo:**

Comparar as medidas acústicas de Cepstral Peak Prominence Smoothed (CPPS) e Acoustic Voice Quality Index (AVQI) de crianças com vozes normais e alteradas, relacionar com o julgamento perceptivo-auditivo (JPA) da voz e estabelecer pontos de corte.

**Método:**

Gravações vocais das tarefas de vogal sustentada e contagem de números de 185 crianças foram selecionadas em um banco de dados e submetidas a análise acústica com extração das medidas de CPPS e AVQI, e ao JPA. O JPA foi realizado individualmente para cada tarefa e as amostras foram classificadas posteriormente como normal ou alterada, e para as tarefas em conjunto definindo-se se a criança passaria ou falharia em uma situação de triagem vocal.

**Resultados:**

Crianças com JPA alterado e que falharam na triagem apresentaram valores menores de CPPS e maiores de AVQI, do que as com JPA normal e que passaram na triagem. O JPA da tarefa de vogal sustentada se relacionou ao CPPS e AVQI, e da tarefa de contagem de números relacionou-se apenas ao AVQI e CPPS números. Os pontos de corte que diferenciam crianças com e sem desvio vocal são 14,07 para o CPPS vogal, 7,62 para o CPPS números e 2,01 para o AVQI.

**Conclusão:**

Crianças com JPA alterado apresentaram maiores valores de AVQI e menores valores de CPPs. O JPA da tarefa de vogal previu todas as medidas acústicas, porém, de contagem previu apenas as medidas extraídas dela. As três medidas foram semelhantes na identificação de vozes sem desvio e vozes disfônicas.

## INTRODUÇÃO

Alteração na qualidade vocal é considerada um sintoma comum na infância e a ocorrência de problemas vocais varia entre 6 e 37% na população pediátrica^(1,2)^. Ela pode afetar negativamente a vida da criança nos aspectos de saúde geral, desenvolvimento socioeducacional, eficiência comunicativa e participação em atividades escolares em grupo^(1,3)^. Sua etiologia é multicausal, podendo ser orgânica, comportamental ou ainda estar relacionada a fatores emocionais^(1,4)^.

Um dos desafios da clínica vocal pediátrica é que as crianças apresentam características anatomofisiológicas diferentes dos adultos. A laringe infantil apresenta imaturidade neuromuscular, com proporção glótica de 1,0; as camadas da lâmina própria das pregas vocais são indiferenciadas e o ligamento vocal é imaturo; os tecidos são vascularizados, com tendência a edema, além do trato vocal ser encurtado^(5-8)^. A presença de instabilidade, soprosidade e rugosidade é esperada exclusivamente em decorrência do desenvolvimento infantil, sem que haja a presença de um distúrbio vocal^(5-8)^.

A avaliação vocal na clínica fonoaudiológica é multidimensional^(9)^. Os procedimentos recomendados são a autoavaliação e avaliação parental, julgamento perceptivo-auditivo (JPA), avaliação aerodinâmica, análise visual e estrutural da laringe, além da análise acústica^(9)^.

A análise acústica está evoluindo no decorrer do tempo e vem-se observando a substituição de medidas tradicionais como os índices de perturbação de frequência, *jitter e shimmer*, por medidas mais robustas como o *Acoustic Vocal Quality Index (*AVQI), uma medida multiparamétrica que avalia várias características ou medidas cepstrais, que independem da extração da frequência fundamental, como a Proeminência do Pico Cepstral Suavizado (*Central Peak Prominence Smoothed*- CPPS)^(5,10,11)^. Levando-se em conta que a voz deve ser avaliada de forma multidimensional, a extração de parâmetros isolados pode ser insuficiente para caracterizá-la^(12)^. O CPPS atualmente é a principal medida acústica para utilização na avaliação clínica vocal de acordo com a recomendação da *American Speech-Language-Hearing Association* – ASHA^(9)^.

O CPPS avalia a periodicidade das emissões vocais e é uma variação da Proeminência do Pico Cepstral (CPP). Essa medida apresenta uma melhor acurácia e estabilidade na extração, permitindo a análise não apenas da emissão de uma vogal sustentada, mas também do sinal de fala. O CPPS suaviza as flutuações no curto prazo no sinal e busca identificar a presença do pico cepstral, um marcador importante da periodicidade da emissão. É uma medida expressa em decibels (dB) e é utilizada em estudos de voz e fala, bem como em aplicações clínicas para avaliar distúrbios vocais, contribuindo para um diagnóstico mais preciso e eficiente^(13-16)^.

Já o AVQI é um índice multiparamétrico, isto é, que utiliza diversos parâmetros para fornecer um escore único da qualidade vocal. Ele considera as tarefas de vogal e contagem de números juntas, e, portanto, tem melhores chances de refletir de modo mais preciso a qualidade vocal do indivíduo^(12)^. Além disso, o AVQI tem mostrado boa acurácia diagnóstica^(15)^, sendo uma ferramenta adequada para determinar a presença de distúrbios vocais.

As tarefas de fala utilizadas para o JPA na clínica vocal envolvem emissões sustentadas, encadeadas e espontâneas^(17)^. Para a população adulta, a emissão sustentada pode ser avaliada como mais desviada em relação às tarefas de fala^(18)^. Assim, sabe-se que as características da voz de uma mesma pessoa possuem variação a depender do tipo de tarefa solicitada^(19)^.

Essas medidas são de extrema relevância clínica, sendo consideradas um componente importante da avaliação clínica vocal de rotina com crianças. Atualmente não há no português brasileiro trabalhos que determinem se para vozes infantis, considerando toda a sua complexidade, essas medidas conseguem diferenciar crianças com vozes normais e alteradas, e se alguma tarefa é mais relevante para essa diferenciação. Além disso, faz-se necessário compreender a relação com o JPA, e estabelecer valores de corte que facilitem a interpretação clínica dos valores de CPPS e AVQI com vozes pediátricas.

Diante disso, o objetivo da pesquisa é comparar as medidas acústicas de CPPS e AVQI de crianças com vozes normais e alteradas, relacionar com o JPA da voz e estabelecer pontos de corte entre essas duas categorias.

## MÉTODO

Trata-se de um estudo observacional, transversal e analítico. O estudo foi aprovado pelo Comitê de Ética da instituição sob o número 2179.073/2010-03. Os pais ou responsáveis pelas crianças assinaram o Termo de Consentimento Livre e Esclarecido (TCLE). As crianças de 12 anos assinaram, ainda, o Termo de Assentimento Livre e Esclarecido (TALE).

A amostra foi constituída por dados e vozes de participantes extraídos de um banco de dados coletados em pesquisa anterior. Os participantes eram crianças recrutadas em um hospital universitário vinculado a uma universidade federal, no momento em que aguardavam por consultas médicas eletivas na área de pediatria (geral, dermatologia, endocrinologia, entre outros), pelo Sistema Único de Saúde (SUS). A amostra foi coletada por conveniência para pesquisa que buscava investigar vozes infantis da população geral.

Foram adotados como critérios de inclusão para a presente pesquisa: crianças pré-púberes, com confirmação por meio de avaliação médica em relação ao estadiamento puberal; ambos os sexos; idade mínima de três anos. Como critérios de exclusão foram considerados: resfriados ou obstruções agudas de vias aéreas por quaisquer motivos no dia da coleta de dados; queixas auditivas; histórico de outros problemas de saúde com impacto na voz. Foram elegíveis para a pesquisa 185 crianças; 93 meninos e 92 meninas, com idades entre 3 e 12 anos (média 6,86 ± 2,2).

O material vocal utilizado do banco de vozes compreendeu a emissão da vogal sustentada “é” e a contagem de números de um a dez, em pitch e loudness habituais. As amostras foram gravadas no programa VOXMETRIA® (CTS Informática, versão 2.5) em arquivo de som *wave*, em notebook da marca Dell®, com microfone unidirecional de cabeça, da marca Karsect modelo HT-9, acoplado à interface de som Andrea Pure Áudio. O microfone foi posicionado a aproximadamente um centímetro do canto da boca do participante (posição diagonal). Caso houvesse dificuldade de calibração em relação à intensidade apresentada pela criança (muito forte ou muito fraca), o microfone era reposicionado em local onde o ganho fosse adequado, o que compreende nível suficiente de sinal, ao redor de dois terços da janela de áudio, como indicado no programa VOXMETRIA® (CTS Informática, versão 2.5).

Para esta pesquisa, os arquivos de áudio extraídos do banco foram editados, desprezando-se os trechos iniciais e finais das gravações das amostras de vogal, que geralmente correspondem a um período de instabilidade natural da voz, mantendo-se o trecho médio, com duração entre três e quatro segundos. As amostras foram editadas, eliminando-se os trechos de silêncio entre as emissões nas amostras de contagem de números.

Os arquivos de áudio foram gravados utilizando o programa computadorizado Audacity e, devido a pequenas diferenças na intensidade do sinal durante a captação das vozes, tanto de vogais quanto de números, as amostras foram padronizadas por calibração manual, no programa Audacity® (versão 2.0.3). Para calibrar os sinais de voz, considerou-se X como sendo o valor absoluto máximo de cada sinal (ou a norma infinita do vetor contendo as amostras do sinal). Em seguida, multiplicou-se a amostra de cada sinal pelo recíproco de X (ou 1/X). Como cada amostra pode assumir valores entre −1 e +1, onde 1 é associado ao nível de volume máximo, este procedimento amplifica o sinal linearmente para apenas aumentar o volume do sinal, sem alterar suas propriedades espectrais e temporais.

Em seguida, as amostras vocais foram submetidas à análise acústica para extração de CPPS e AVQI, por meio do software PRAAT, versão 6.06. Para a extração do CPPS, foram utilizados os comandos específicos para obtenção das medidas em falantes do português brasileiro (PB), realizando-se uma extração para a tarefa de vogal e uma extração para a tarefa de contagem de números. Para a obtenção do AVQI, foi utilizado um script com a versão de número 03.01, com a utilização simultânea das amostras de tarefa de vogal e tarefa de contagem de números^(13)^.

O JPA foi realizado por três fonoaudiólogos especialistas em voz, todos com mais de dez anos de experiência clínica e científica na área de voz, e com experiência em disfonia infantil, que não conheciam previamente a pesquisa. Os juízes analisaram as amostras por consenso, ou seja, estiveram juntos, em um mesmo ambiente, e ouviram as vozes sob as mesmas condições, em ambiente silencioso e reproduzidas em caixa de som de qualidade profissional. Foram analisadas separadamente as amostras referentes à tarefa de vogal e à tarefa de números. Os juízes deveriam ouvir a amostra de cada tarefa, quantas vezes necessário, e atribuir o grau geral de desvio vocal (G) por meio de escala numérica de quatro pontos, considerando que zero indica ausência de desvio, um indica desvio discreto, dois indica desvio moderado e três indica desvio intenso. O julgamento perceptivo-auditivo foi realizado em aproximadamente duas horas, em um único dia. A concordância interna da análise dos juízes feita por consenso foi analisada por meio do teste de Kappa com a repetição de aproximadamente 10% das amostras, com valor de Kappa de 0,75.

Para a análise dos dados na presente pesquisa, prosseguiu-se com a classificação das amostras vocais da seguinte forma: as amostras classificadas pelos juízes com desvios ausentes (zero) ou discretos (um), foram consideradas normais; as amostras classificadas pelos juízes com desvios moderados (dois) ou intensos (três), foram consideradas alteradas. Na sequência, para se estabelecer um resultado único de cada criança, referente a uma possível situação de triagem vocal, prosseguiu-se da seguinte forma: as crianças que tiveram as vozes consideradas normais em ambas as tarefas (vogal e contagem de números) ou que tiveram apenas uma das tarefas considerada alterada foram classificadas como aquelas que passariam em uma situação de triagem vocal; as crianças que tiveram as vozes consideradas como alteradas em ambas as tarefas (vogal e contagem de números) foram classificadas como aquelas que falhariam em uma situação de triagem vocal. Esclarece-se que todas as crianças da amostra tiveram diferença de no máximo um grau entre as tarefas de fala.

Os dados foram analisados de forma descritiva e inferencial. Utilizou-se o software SPSS 25.0. Na análise descritiva das variáveis quantitativas foram calculadas as medidas de tendência central (média e mediana), variabilidade (desvio padrão) e posição (mínimo, máximo, primeiro e terceiro quartis). Na análise descritiva das variáveis qualitativas nominais foram calculadas a frequência absoluta e a frequência relativa percentual.

No presente estudo, as medidas acústicas foram as variáveis dependentes, e o JPA e a triagem vocal foram as variáveis independentes. A comparação das medidas acústicas em função do JPA e da triagem vocal foi realizada com o Teste de Mann-Whitney. A análise preditiva das medidas acústicas foi realizada por meio de análise de regressão linear múltipla, com método backward. Além disso foram realizadas análises de curva ROC para as medidas acústicas, utilizando-se como padrão-ouro o resultado da triagem vocal. Considerou-se um nível de significância de 5%.

## RESULTADOS

Na tarefa de vogal há maior ocorrência de vozes alteradas (n=105; 56,76%) em relação as vozes normais (n=80; 43,24%). Na tarefa de contagem de números existe a ocorrência de 72 vozes alteradas (38,92%) e 113 vozes normais (61,08%). No resultado da triagem há maior frequência de vozes normais do que de amostras alteradas (normais n=128 – 69,19%; alteradas n-57; 30,81%).

Visualiza-se nas Tabelas 1 e 2 que as crianças que obtiveram classificação das tarefas de vogal e de contagem de números como alteradas no JPA apresentaram valores inferiores de CPPS vogal (p<0,001; p=0,044) e CPPS números (p=0,001; p<0,001), e valores superiores de AVQI (p<0,001; p<0,001), que as crianças cujas amostras foram classificadas como normais no JPA.

**Tabela 1:** Comparação das variáveis dependentes em função da variável independente classificação JPA vogal

Variável	JPA Vogal	Média	DP	Mínimo	Máximo	1Q	Mediana	3Q	p-valor
CPPS vogal	Normal	14,71	1,70	10,50	19,01	13,36	14,61	15,75	0,000
	Alterada	12,95	1,61	9,03	17,76	11,87	12,66	14,22	
CPPS números	Normal	8,34	1,42	5,78	12,45	7,28	8,28	9,19	0,001
	Alterada	7,63	1,26	4,95	10,68	6,96	7,53	8,36	
AVQI	Normal	1,24	0,98	-1,70	5,04	0,69	1,23	1,78	0,000
	Alterada	2,06	0,89	-0,11	4,02	1,58	2,15	2,65	

Teste de Mann-Whitney

Legenda: DP = desvio padrão; 1Q = primeiro quartil; 3Q = terceiro quartil

**Tabela 2:** Comparação das variáveis dependentes em função da variável independente classificação JPA números

Variável	JPA Números	Média	DP	Mínimo	Máximo	1Q	Mediana	3Q	p-valor
CPPS vogal	Normal	13,93	1,93	9,36	19,01	12,48	14,11	15,00	0,044
	Alterada	13,37	1,71	9,03	17,76	12,24	13,13	14,47	
CPPS números	Normal	8,29	1,31	5,78	12,45	7,28	8,17	9,22	0,000
	Alterada	7,38	1,29	4,95	11,65	6,62	7,30	8,12	
AVQI	Normal	1,46	0,99	-1,70	5,04	0,77	1,46	2,14	0,000
	Alterada	2,08	0,93	-0,63	4,02	1,55	2,21	2,69	

Teste de Mann-Whitney

Legenda: DP = desvio padrão; 1Q = primeiro quartil; 3Q = terceiro quartil

A Tabela 3 mostra que as crianças que falharam na triagem vocal apresentaram valores inferiores de CPPS vogal (p<0,001) e CPPS números (p<0,001), e valores superiores de AVQI (p<0,001), quando comparadas às crianças que passaram na triagem.

**Tabela 3:** Comparação das variáveis dependentes em função da variável independente triagem vocal

Variável	Triagem	Média	DP	Mínimo	Máximo	1Q	Mediana	3Q	p-valor
CPPS vogal	Passou	14,03	1,91	9,36	19,01	12,80	14,12	15,15	<0,001
	Falhou	12,99	1,54	9,03	17,76	12,02	12,66	14,31	
CPPS números	Passou	8,24	1,35	5,78	12,45	7,25	8,15	9,18	<0,001
	Falhou	7,25	1,17	4,95	10,06	6,57	7,24	8,08	
AVQI	Passou	1,44	0,99	-1,70	5,04	0,72	1,45	2,14	<0,001
	Falhou	2,31	0,78	0,10	4,02	2,05	2,34	2,74	

Teste de Mann-Whitney

Legenda: DP = desvio padrão; 1Q = primeiro quartil; 3Q = terceiro quartil

Foram desenvolvidos três modelos preditivos para verificar se as variáveis independentes JPA das tarefas de vogal e contagem de números eram preditivas das variáveis dependentes AVQI, CPPS vogal e CPPS números (Tabela 4). As variáveis independentes JPA da vogal (β = 0,338; t = 4,724; p = 0,000 ; β = -0,164; t = -2,208; p = 0,029) e JPA da contagem de números (β = 0,177; t = 2,474; p = 0,014 ; β = -0,262; t = -3,524; p = 0,001; respectivamente) foram previsoras das variáveis dependentes AVQI [F(2, 182) = 21,200; p <0,001; R2 = 0,189] e CPPS números [F(2, 182) = 13,174; p <0,001; R2 = 0,117]. A variável independente JPA da vogal (β = -0,471; t = -7,219; p <0,001) foi previsora da variável dependente CPPS vogal [F(1, 183) = 52,119; p<0,001; R2 = 0,222].

**Tabela 4:** Modelo preditivo das variáveis dependentes AVQI, CPPS vogal e CPPS números

Modelo		Coeficientes não padronizados		Coeficientes padronizados	t	p-valor
		B	Erro padrão	Beta		
AVQI	(Constante)	1,172	0,106		11,031	0,000
	Vogal	0,689	0,146	0,338	4,724	0,000
	Números	0,367	0,148	0,177	2,474	0,014
CPPS vogal	(Constante)	14,715	0,185		79,742	0,000
	Vogal	-1,768	0,245	-0,471	-7,219	0,000
CPPS números	(Constante)	8,481	0,150		56,621	0,000
	Números	-0,737	0,209	-0,262	-3,524	0,001
	Vogal	-0,454	0,206	-0,164	-2,208	0,029

Regressão linear múltipla, método stepwise

Variáveis independentes: vogal, números

Legenda: t = estatística T

Os resultados mostram uma curva (Figura 1) estatisticamente significativa para o AVQI (AUC = 0,76; EP = 0,04; p <0,001; IC 95% = 0,69 – 0,84), demonstrando que, ao serem escolhidos aleatoriamente, 76% dos casos que falharam na triagem vocal apresentaram valores maiores no AVQI, do que os casos que passaram na triagem vocal. O ponto de corte que maximiza a sensibilidade e a especificidade foi 2,05 (i.e. abaixo de 2,0 e acima de 2,1), com sensibilidade 0,75 e especificidade 0,72. O AVQI possui capacidade semelhante para identificar vozes desviadas, e vozes sem desvio.

**Figura 1:** Curva ROC AVQI

Para o CPPS vogal também foi observada uma curva (Figura 2) estatisticamente significativa (AUC = 0,78; EP = 0,03; p <0,001; IC 95% = 0,715 - 0,846), demonstrando que, ao serem escolhidos aleatoriamente, 78% dos casos com desvio vocal no JPA da amostra de vogal apresentaram valores menores no CPPS vogal, do que os casos sem desvio vocal. O ponto de corte que maximizou a sensibilidade e a especificidade foi 14,07 (i.e. abaixo de 14,0 e acima de 14,1), com sensibilidade 0,73 e especificidade 0,66. O CPPS vogal possui capacidade semelhante para identificar vozes desviadas, e vozes sem desvio.

**Figura 2:** Curva ROC CPPs Vogal

Para o CPPS números também foi obtida uma curva (Figura 3) estatisticamente significativa (AUC = 0,70; EP = 0,04; p<0,001; IC 95% = 0,62 – 0,78), demonstrando que, ao serem escolhidos aleatoriamente, 70% dos casos com desvio vocal no JPA da contagem de números apresentaram valores menores do que os casos sem desvio vocal no CPPS números. O ponto de corte que maximizou a sensibilidade e a especificidade foi 7,62 (i.e. abaixo de 7,6 e acima de 7,7), com sensibilidade 0,63 e especificidade 0,68. O CPPS números possui capacidade semelhante para identificar vozes desviadas, e vozes sem desvio.

**Figura 3:** Curva ROC CPPs Números

## DISCUSSÃO

No presente estudo, foram altas as frequências relativas de vozes alteradas no JPA, considerando uma situação de triagem vocal (30,81%), que associou a análise tanto da emissão sustentada quanto da emissão encadeada. Ainda assim, tais valores estão em consonância com a literatura no que se refere à ocorrência de problemas vocais na população pediátrica^(1,2)^.

Na tarefa de vogal há maior ocorrência de vozes alteradas (n=105; 56,76%) em relação as vozes normais (n=80; 43,24%). Na tarefa de contagem de números existe a ocorrência de 72 vozes alteradas (38,92%) e 113 vozes normais (61,08%). No resultado da triagem há maior frequência de vozes normais do que de amostras alteradas (normais n=128 – 69,19%; alteradas n-57; 30,81%).

No presente estudo, pôde-se observar que as medidas cepstrais AVQI e CPPS diferenciaram crianças com vozes normais e alteradas pelo JPA, independentemente de a amostra ser de fala sustentada, encadeada ou ambas. Os resultados obtidos corroboram a literatura^(9,14,15)^, mostrando que crianças saudáveis apresentam maiores valores de CCPS e menores valores de AVQI, que crianças com vozes alteradas.

Por meio da análise dos modelos de regressão utilizados no presente estudo, foi possível concluir que o JPA da vogal sustentada se relacionou às três medidas, enquanto o JPA da contagem de números relacionou-se apenas ao AVQI e ao CPPS números. Sabe-se que a tarefa vocal contribui para a variabilidade do JPA, visto que, na contagem de números, é possível observar variações temporais e espectrais causadas pelo início e final da palavra, pausas, fonemas surdos, contexto fonético, flutuações prosódicas na frequência e intensidade, velocidade de fala, entre outros. Já na vogal sustentada, existem condições subglótica e supraglótica relativamente constantes^(19)^.

Com crianças em situações de triagem, as crianças com lesão laríngea tendem a falhar tanto em emissões sustentadas quanto encadeadas, diferentemente de crianças com laringe normal, que podem eventualmente falhar em apenas uma das tarefas^(7)^. Em vozes de adolescentes, a vogal sustentada é a tarefa capaz de identificar as instabilidades vocais típicas do período de muda vocal, não verificadas nas tarefas de contagem de números ou leitura de texto^(18)^.

Acredita-se que em crianças, que também possuem instabilidade como um desvio esperado devido a imaturidade neuromuscular^(20)^, e soprosidade relacionada à fenda glótica posterior devido principalmente à configuração anatômica típica da cartilagem cricóidea nessa faixa etária^(6)^, a tarefa de vogal também seja mais sensível para identificação de desvios vocais, sendo portanto, preditiva de todas as medidas acústicas multiparamétricas. Faz-se importante mencionar, ainda, que os resultados obtidos na análise da curva ROC para as variáveis AVQI e CPPS vogal corroboram os achados de estudos realizados com crianças, os quais evidenciam o uso das medidas cepstrais como indicadores de desvio vocal^(9,15)^.

No presente estudo, o AVQI apresentou maiores valores de sensibilidade, diferentemente de um estudo com vozes pediátricas cujo índice apresentou maiores valores de especificidade^(15)^. No entanto, a referida pesquisa**^(16)^** estipulou um valor de corte de 3,46, superior ao obtido no presente estudo (2,05), o que facilita a exclusão de um quantitativo maior de crianças sem desvio vocal.

Há influência da língua falada na definição das medidas de corte^(21)^ e é importante considerar que na população adulta falante de português brasileiro, cujo valor de corte é de 1,33^(12)^, os valores também se encontram inferiores aos obtidos nas populações de outros países, evidenciando que tal aspecto também pode ter influência de questões linguísticas e culturais^(22)^.

O valor de corte superior em crianças que em adultos falantes do português brasileiro pode ser atribuído aos desvios esperados na qualidade vocal pediátrica pelo próprio processo de desenvolvimento^(23)^, e que exigem, portanto, parâmetros e análises diferenciadas^(24)^. Além disso, corrobora resultados de recente estudo, realizado com a população pediátrica, adulta e idosa de um mesmo país e evidenciou que os valores de corte de AVQI que diferenciam vozes normais e desviadas são superiores na população infantil quando comparados aos obtidos pela população adulta^(24)^.

No que se refere ao CPPS, diferentemente do presente estudo, pesquisa anterior obteve maior sensibilidade no CPP números para diferenciar vozes normais e desviadas^(9)^. Pode-se entender que esta medida também seja influenciada pelas questões linguísticas dos diferentes idiomas, o que a torna mais complexa para comparação de populações de diferentes países.

No que se refere aos valores de corte obtidos na presente pesquisa para a diferenciação de vozes infantis normais e alteradas (CPPS vogal 14,07 e CPPS números 7,62), estudos anteriores também obtiveram maiores valores de CPP com tarefa de vogais em relação ao CPP com tarefa de contagem de números^(25,26)^. Não foram localizados estudos com crianças que tenham estabelecido valores de corte do CPPS. No entanto, é importante mencionar que estudo que avaliou crianças e adolescentes de 5 a 18 anos, sendo 128 do sexo feminino e 131 do sexo masculino, falantes do português brasileiro, obteve valores médios de CPPs semelhantes aos obtidos no valor de corte do presente estudo^(27)^. A pesquisa supracitada dividiu crianças e adolescentes por faixa etária e sexo e, nas faixas etárias que compreenderam idades entre 5 e 11 anos, as médias de CPPs para amostras de vogal variaram entre 13,994 e 15,203. Destaca-se ainda que estudo realizado com medida similar, o CPP, concluiu que as medidas cepstrais são úteis na diferenciação de crianças saudáveis e disfônicas^(9)^.

É importante mencionar que os valores de corte de CPPS vogal e CPPS números obtidos no presente estudo também são inferiores aos valores comumente obtidos na população adulta^(28)^, o que é esperado, conforme supracitado, em decorrência do desvio vocal relacionado ao processo de desenvolvimento^(26)^.

O presente estudo apresentou limitação com relação ao material da tarefa de contagem de números, que foi de 1 a 10, diferentemente da versão do AVQI validada no Português Brasileiro que utilizou contagem de números de 1 a 11 na fala encadeada. Porém, sabe-se que a influência da contagem menor não é um fator relevante^(12)^.

Além disso, aponta-se como limitação a não subdivisão das crianças por faixa etária. Embora alguns estudos apontem que não há mudanças nos resultados das medidas acústicas de perturbação e ruído com o avanço da idade em crianças^(29,30)^, em estudos recentes sobre medidas cepstrais, com crianças e adolescentes de faixa etária mais ampla, evidenciaram-se algumas diferenças relacionadas ao gênero^(27)^ e tipo de tarefa^(14)^.

Nesse sentido, considerando que o desenvolvimento vocal infantil é um fenômeno complexo, sugere-se que novos estudos considerem a referida subdivisão para elucidar a distribuição das medidas de CPPS e AVQI. Reforça-se, no entanto, que apenas crianças comprovadamente pré-púberes participaram deste estudo, sendo avaliadas pelo médico pediatra no dia da coleta dos dados vocais. Tratando-se do primeiro estudo de medidas cepstrais e índices multiparamétricos na infância, optou-se por oferecer dados abrangentes de pré-púberes, sem subdivisões.

Acredita-se que os dados do presente estudo serão úteis para caracterização e interpretação dos valores encontrados na prática clínica e na pesquisa para medidas multiparamétricas com crianças. Além disso, tanto o AVQI quanto o CPPS podem ser usados como parâmetros objetivos na avaliação e no acompanhamento terapêutico da população pediátrica em acompanhamento fonoaudiológico.

## CONCLUSÃO

Crianças com desvio vocal apresentaram maiores valores de AVQI e menores valores de CPPs, do que as crianças sem desvio vocal avaliado auditivamente, independentemente da tarefa de fala. O JPA da tarefa de vogal sustentada prevê todas as medidas acústicas, porém, o JPA da tarefa de contagem de números foi preditivo apenas das medidas extraídas dele, a saber o AVQI e CPPS. Os pontos de corte que diferenciam crianças com e sem desvio vocal são 14,07 para o CPPS vogal, 7,62 para o CPPS números e 2,01 para o AVQI. As três medidas foram semelhantes na identificação de vozes sem desvio e vozes disfônicas.
